# Detecting Weak Signals by Combining Small P-Values in Genetic Association Studies

**DOI:** 10.3389/fgene.2019.01051

**Published:** 2019-11-20

**Authors:** Olga A. Vsevolozhskaya, Fengjiao Hu, Dmitri V. Zaykin

**Affiliations:** ^1^Department of Biostatistics, College of Public Health, University of Kentucky, Lexington, KY, United States; ^2^Biostatistics and Computational Biology, National Institute of Environmental Health Sciences, National Institutes of Health, Research Triangle Park, NC, United States

**Keywords:** combining evidence, rank truncated product, a rank truncated product RTP, augmented rank truncation, adaptive augmented rank truncation

## Abstract

We approach the problem of combining top-ranking association statistics or P-values from a new perspective which leads to a remarkably simple and powerful method. Statistical methods, such as the rank truncated product (RTP), have been developed for combining top-ranking associations, and this general strategy proved to be useful in applications for detecting combined effects of multiple disease components. To increase power, these methods aggregate signals across top ranking single nucleotide polymorphisms (SNPs), while adjusting for their total number assessed in a study. Analytic expressions for combined top statistics or P-values tend to be unwieldy, which complicates interpretation and practical implementation and hinders further developments. Here, we propose the augmented rank truncation (ART) method that retains main characteristics of the RTP but is substantially simpler to implement. ART leads to an efficient form of the adaptive algorithm, an approach where the number of top ranking SNPs is varied to optimize power. We illustrate our methods by strengthening previously reported associations of μ-opioid receptor variants with sensitivity to pain.

## Introduction

Complex diseases are influenced by multiple environmental and genetic risk factors. A specific factor, such as a single mutation, may convey a high risk, but population frequencies of high risk factors are usually low, and substantial contribution to disease incidence can be attributable to accumulation of multiple but weak determinants within individuals. Genetic determinants of complex diseases that had been identified by genetic association studies tend to carry modest effects; yet, power to detect such variants, as well as accuracy of identifying individuals at risk, can be improved by combining multiple weak predictors. The main challenge in detecting specific variants is low statistical power, but the overall accumulated effect of many individually weak signals can be much stronger. It is convenient to combine statistical summaries of associations—for example, P-values, and this approach can be nearly as efficient as analysis of raw data (Lin and Zeng, 2010). In observational research, methods for combining P-values are commonly associated with meta-analyses that pool results of multiple experiments studying the same hypothesis. The combined P-value then aggregates signals across all *L* studies, potentially providing a higher level of assurance that the studied risk factor is associated with disease. Furthermore, if samples in those studies are taken from populations that are similar with respect to the effect size magnitude, the combined meta-analytic P-value will well approximate the one that would have been obtained by pooling together all raw data and performing a single test (Zaykin, 2011).

P-values can also be combined when the *L* hypotheses are distinct, and when the interest is in detecting the overall signal. Such applications are common and include gene set and pathway analyses. Specifically, a typical strategy in computation of gene- and pathway-scores includes (1) mapping individual SNPs to genes, followed by combining their association P-values into gene-scores, and (2) grouping genes into pathways and combining gene-scores into pathway-scores.

Existing tools for combining P-values (*P_i_,i* = 1, …, *L*) are often based on the sum of *P_i_*‘s transformed by some function *H*. For example, Fisher (1932) test is based on the log-transformed P-values, *H*(*P_i_*)=–21n(*P_i_*), which are then added up to form a test statistics $T=\sum_{i=1}^{L}H(P_{i})\sim \chi_{\left(2L\right)}^{2}$ , where $\chi_{\left(2L\right)}^{2}$ has the chi-square distribution with 2 *L* degrees of freedom. When a portion of *L* distinct associations is expected to be spurious, it is advantageous to combine only some of the predictors using a truncated variation of combined P-value methods or emphasize the smallest P-values. For instance, Zaykin et al. (2002) proposed the truncated product method (TPM) as a variation of the Fisher test, which was trimmed by the indicator function, *I*(*P_i_*≤ α), that is equal to zero if *P_i_*> α, and one if *P_i_*≤ α; 0 < α ≤ 1 is a truncation threshold. The combined P-value, *P*_TPM_, is then given by the cumulative distribution function (CDF) of $W=\sum_{i=1}^{L}\ln(P_{i})I(P_{i}\leq \alpha )$ . With the TPM approach, the threshold α is fixed while the number of P-values that form the sum *W* is random. Similarly inspired methods include the “tail strength measure” Taylor and Tibshirani (2005) and a method by Jiang et al. (2011).

A related popular method for combining top-ranking P-values is the rank truncated product (RTP) (Zaykin, 2000; Dudbridge and Koeleman, 2003; Zaykin et al., 2007a). In RTP, the number of P-values to be combined, *k*, is fixed, rather than the P-value threshold, as in TPM. The resulting combined P-value can be found from the cumulative distribution of the product:

$$P_{\text{RPT}}=\text{Pr}\left\{\prod_{In=1}^{k}P_{\left(i\right)}\leq w\right\}=1-\text{Pr}\left\{\sum_{i=1}^{k}\ln\left[P_{\left(i\right)}\right]>\ln\left[w\right]\right\},$$

where *P*_(i)_ is the *i*th smallest P-value, *i* = 1,…,*k*. RTP leads to an appealing extension, where *k* can be chosen adaptively, to maximize statistical power (Hoh et al., 2001; Yu et al., 2009; Zhang et al., 2013). Adaptive rank truncated product (aRTP) variations optimize selection of the truncation point *k* among all (or a subset) of possible values 1 ≤ *k* ≤ *L*. Adaptive extensions for TPM are not as straightforward because the threshold α is a continuous variable, but one can resort to evaluating the distribution over a set of grid points (Sheng and Yang, 2013). In adaptive extensions of TPM and RTP, the final test statistic is the minimum P-value observed at various candidate truncation points.

**Figure 1:**
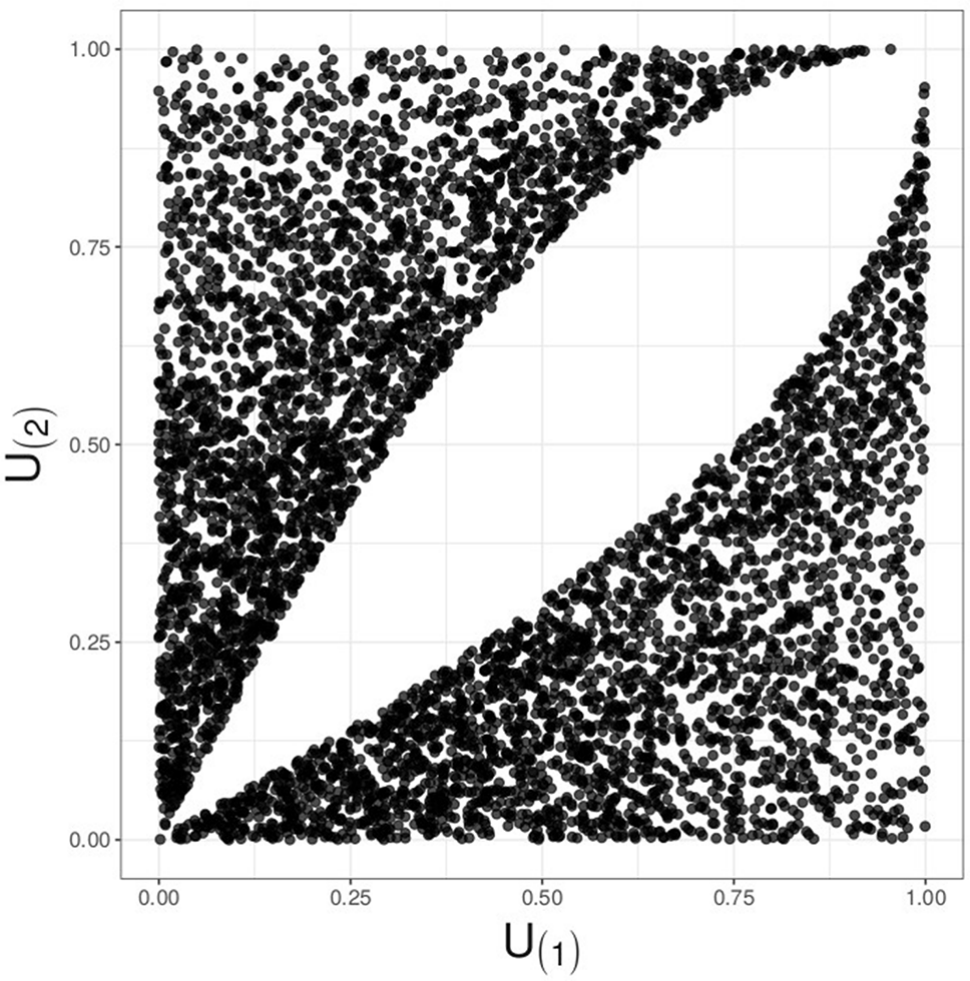
Illustration for decorrelated yet dependent P-values; *k* = 2, *L* = 4. A plot of simulated and decorrelated values, *U*_(_*_1_*_)_*vs.U*_(_*_2_*_)_, reveals a hole in the middle, instead of the complete Malevich black square, indicating dependency.

The RTP null distribution is considerably more complicated than that of TPM. Complexity of the RTP distribution is due to dependency between ordered P-values. When *k = L*, this dependency is inconsequential because a statistic is formed as a sum of *L* terms, and its value does not change if the terms are re-ordered. In fact, when *k = L*, the RTP P-value is the same as the Fisher combined P-value, derived *via* a CDF of a sum of independent chi-square variables. However, if 1 < *k* < *L*, the *k* smallest P-values remain correlated and dependent even if these *k* values are randomly shuffled. The dependency is induced through *P*_(k+1)_ being a random variable: when *P*_(_*_k+1_*_)_ happens to be relatively small, the *k* P-values have to squeeze into a relatively small interval from zero to that value. This induces positive dependency between random sets of *k* smallest P-values, similar to the clustering effect in random effects models. Although the linear correlation can be eliminated by scaling the largest P-value, *P*_(_*_k_*_)_, the *k* values remain dependent, as illustrated in **Figure 1** (see **Supplementary Material S-1** for discussion).

Applications of combining independent P-values remain important in statistical research, and there is clear preference among practitioners for methods that are based on simple and transparent approaches, such as the Fisher or the inverse normal (Stouffer’s) tests (Fisher, 1932; Stouffer et al., 1949; Loughin, 2004; Whitlock, 2005; Zaykin, 2011; Won et al., 2009). Here, we derive a simple, easily implemented theoretical form of the RTP distribution for independent P-values which further leads to derivation of a new statistic. The new statistic, which we call the augmented RTP, or ART, is based on the product of the first smallest P-values, just like the RTP, but, unlike the RTP, the distribution of the new statistic is given by standard functions, and its computation avoids explicit integration. Despite simplicity, ART is at least as powerful as RTP, according to our simulation studies. Moreover, the ART leads to an adaptive statistic, where the number of the smallest P-values to combine can be determined analytically to maximize power. Next, we extend our methods by allowing dependence in the observed P-values. In genetic association studies, P-values are often correlated due to linkage disequilibrium (LD). The LD correlation is typically accounted for through permutational or other resampling approaches, where P-values are simulated under the null hypothesis while preserving LD between genetic variants. While such approaches are practical and easy to implement, it is also possible to de-correlate P-values before combining them and then use any of the approaches developed under the independence assumption. Surprisingly, we find that the decorrelation step often improves power. In particular, we find that, when association with disease is markedly different among variants within a gene, statistical power of standard methods (without the decorrelation step) approaches a plateau as a function of LD and does not improve as the number of SNPs increases. In contrast, power of our proposed decorrelation method increases steadily with the number of SNPs. Our analytical results as well as simulation experiments demonstrate this property for both ART (where *k* is chosen beforehand and fixed) and for the adaptive variations of RTP and ART (aRTP and ART-A). Finally, we illustrate usefulness of the proposed methods by strengthening an overall, gene-based association *via* combining previously reported P-values between pain sensitivity and individual SNPs within the μ-opioid receptor.

## Material and Methods

### Theoretical RTP Distribution and Augmented RTP, the ART

Even when P-values are independent, previously proposed theoretical forms of the RTP distribution are cumbersome and result in expressions that involve repeated integration (Zaykin, 2000; Dudbridge and Koeleman, 2003; Nagaraja, 2006; Zaykin et al., 2007b). For example, Nagaraja (2006) gives the cumulative distribution for the statistic $W_{k}=-\sum_{i=1}^{k}\ln P_{\left(i\right)}$ and *k< L*, as:

(1) $$\begin{array}{l} \text{Pr}(W_{k}>w)=\sum_{j=1}^{k}w_{j}\text{exp}\left\{-\frac{c_{j}w}{c_{k+1}}\right\}\frac{1}{(L-K-1)!}\int_{0}^{w}\exp\{yd_{j}\}y^{L-k-1}dy\\ +\sum_{s=o}^{L-k-1}\exp\{-w\}\frac{w^{s}}{s!},\;\text{where}\\ c_{j}=L-j+1,\\ d_{j}=\frac{k+1-j}{L-k},\\ w_{j}=\frac{1}{L-j+1}\frac{L!}{(L-k)!}\frac{{\left(-1\right)}^{k}-j}{(j-1)!(k-j)!} \end{array}$$

Theoretical forms of the RTP distribution (e.g., Eq. 1) may retain order-specific terms. Here, we proceed to a simpler representation by noting that every random realization of *k* smallest P-values can be shuffled. This step does not change the value of the product, *W_k_* (or its logarithm), which is our statistic of interest, but implies that we can treat the joint *k*-variate distribution as governed by the same pair-wise dependence for every pair of variables. Moreover, variables of that shuffled distribution are identical marginally. The dependency is induced completely through randomness of *P*_(_*_k+1_*_)_, and conditionally on its value, the {*W_k_*|*p*_(_*_k+1_*_)_} distribution is given by standard independence results. Then, *P*_RTP_ is given by the marginal CDF of *W_k_*. Based on this conceptual model, we derived the following representation of RTP where a single integral is evaluated in a bounded interval (0,1), which allows one to evaluate the RTP distribution as a simple average of standard functions. Specifically, we derive a simple expression for the RTP distribution as the expectation of a function of a uniform (0 to 1) random variable:

(2) PRTP(k)=Pr⁡(Wk≤w)=1−∫01Gk{ln([Bk+1−1(u)]wk)}duPRTP(k)=E{H(U|k,w)},

where $B_{k+1}^{-1}(\cdot )$ is inverse CDF of Beta(*k*+1,*L*−*k*) distribution, *G_k_*(·) is CDF of Gamma(*k*, 1), and $H(u|k,w)=G_{k}\left(\ln\left(\frac{{\left[B_{k+1}^{-1}(u)\right]}^{k}}{w}\right)\right).$ *P*_RTP_(*k*) is the combined RTP P-value. Notably, given the value of the product of *k* P-values, *W* = *w*, we can simultaneously evaluate *P*_RTP_(*k*+1):

(3) $$\begin{array}{l} P_{\text{RTP}}(k+1)=\\ \text{Pr}(W_{k+1}\leq w)=1-\int_{0}^{1}G_{k}\left\{\ln\left(\frac{{\left[B_{k+1}^{-1}(u)\right]}^{k+1}}{w}\right)\right\}du. \end{array}$$

Details and the derivation are given in **Supplementary Material S-2**.

The conditional independence of *k*−1 smallest P-values, given a value of the beta-distributed *k*-th smallest P-value, leads to a simple statistic which (just as RTP) is a function of the product of the *k*-th smallest P-values. This statistic and its distribution are not an approximation to *W_k_* and the RTP distribution. However, similarly to RTP, the new statistic is designed to capture information contained in the first *k* smallest P-values. Motivation for our augmented RTP method (ART) follows from the intermediate results of the derivation of the RTP distribution. Note that the distribution of *W_k_* conditional on (*k* + 1) involves a product of *k* uniforms and a beta random variable. Moreover, the product and the beta variable are independent. On the one hand, we could have proceeded by dividing every *P*_(_*_1:k_*_)_ value by the observed *p*_(_*_k+1_*_)_ and employed the gamma CDF to $\sum_{i=1}^{k}-\ln(P_{\left(i\right)}/p_{\left(k+1\right)})$ to obtain the combined P-value. However, this approach ignores information contained in *P*_(_*_k+1_*_)_ magnitude. On the other hand, we can exploit the independence of the gamma-distributed sum and *P*_(_*_k+1_*_)_ by transforming *P*_(_*_k+1_*_)_ to a gamma random variable and adding the result to the sum. Specifically, to construct the new statistic, we propose the following transformation that involves the product *W_k−1_* and the variable *P_(k)_*. These transformations yield three independent variables, which are next added together and give a gamma-distributed random variable,

(4) $$\begin{array}{l} A_{k}=\\ -\ln\{W_{k-1}\}+(k-1)\ln\{P_{\left(k\right)}\}+G_{\lambda }^{-1}\{1-B_{k}(P_{\left(k\right)})\}, \end{array}$$

where $G_{k}^{-1}(\cdot )$ is inverse CDF of Gamma (*k*,1),

λ = (*k* – 1) × *E* {– ln (*P_(k)_*)} = (*k* – 1) (Γ′ (*L* + 1)/Γ (*L* + 1) – Γ′ (*k*)*/*Γ (*k*)),

Γ′ is the first derivative of a gamma function; and *B_k_*(*x*) is the CDF of Beta(*k*, *L*−*k*+1) distribution evaluated at *x*. The shape parameter λ is chosen so that the two last terms in Eq. 4 (that are both transformations of *P_(k)_*) have the same expectation. The shape parameter here plays a role of a weight given to the last term. This is similar to the Lancaster method for combining P-values, where the gamma shape parameter (equivalently, the degrees of freedom of a chi-square distribution) has been used to give differential weights to P-values (Lancaster, 1965).

Given the observed value *A_k_*=*a_k_*, the combined P-value is

(5) $$P_{\text{ART}}=\mathrm{Pr}(A_{k}\leq a_{k})=1-G_{k+\lambda -1}(a_{k}).$$

**Figure 2:**
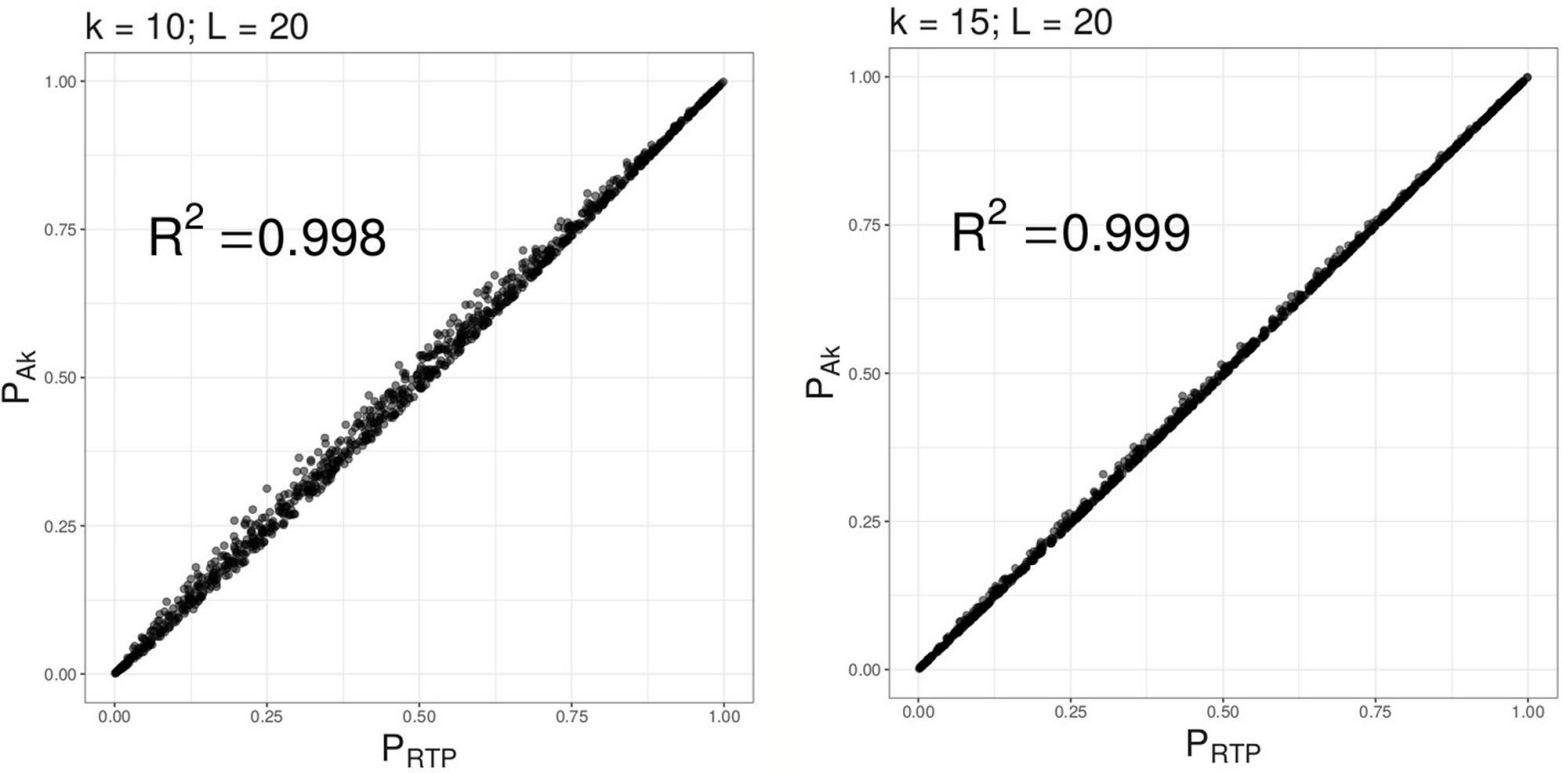
Combined P-values based on A*_k_* *versus* RTP statistic. Multiple combined P-values were computed using the 2 proposed statistics based on either top 10 or top 15 P-values out of *L* = 20 tests.

Under the null hypothesis, as illustrated by **Figure 2**, combined P-values based on the proposed method (ART) are very similar to *P*_RTP_, and approach *P*_RTP_ as *k* increases. However, under the alternative, we find (as described in “*Results*” section) that ART has either the same or higher power than RTP. Furthermore, the combined P-value, ART, can be easily computed in R using its standard functions. A short example and an R code are given in **Supplementary Material S-3**.

### Adaptive ART Method, ART-A

As we discussed earlier in *Introduction*, the number of *k* P-values to be combined by the RTP method (or ART) is fixed and needs to be pre-specified. The choice of *k* is somewhat arbitrary; so, a researcher may wish to evaluate ART at several values of *k*, consequently choosing *k* that corresponds to the smallest combined P-value. However, this additional step creates another layer of multiple comparisons, which needs to be accounted for. Yu et al. (2009) proposed an empirical procedure to evaluate adaptive RTP (aRTP) method based on the minimum P-value computed over various candidate truncation points. To avoid a cumbersome two-level permutation procedure, they built on the method suggested by Ge et al. (2003) to reduce computational time. While computationally efficient, the method requires to store a large *B*×*L* matrix, with every row containing *L* P-values generated under the null distribution over *B* simulated experiments. Zhang et al. (2013) derived analytic but mathematically complex aRTP distribution, which needs to be evaluated using high-dimensional integration. Here, we propose a new and easily implemented version of the theoretical distribution for ART, ART-A. The method exploits the fact that ordered P-values can be represented as functions of the same number of independent uniform random variables (**Supplementary Material S-4**). The two main ideas behind ART-A are first to approximate the Gamma distribution with a large shape parameter by the normal distribution, and second to use the fact that the joint distribution of the partial normal sums follows a multivariate normal distribution.

### Correlated P-Values

We further extend the proposed methods to combine correlated P-values *via* decorrelation by orthogonal transformation approach, DOT. Let *L* correlated P-values, (*p_1_*, *p_2_*, …, *p_L_*) originate from statistics that jointly follow a standard multivariate normal distribution, **y ∼ MVN** (**μ** = **0**, **Σ**), under *H*_0_. For two-sided P-values, the elements of **y** are squared. Elements of the vector of squared variables, $y_{j}^{2}$ , follow the one degree of freedom chi-square distribution with $Cor(y_{i}^{2},y_{j}^{2})=\Sigma_{ij}^{2}$ . Dependent variables can be transformed into independent variables by using eigendecomposition of **Σ**, such that **Σ = QΛQ^−1^**, where **Q** is a square matrix, with *i*th column containing eigenvector **q***_i_* of **Σ**, and **Λ** is the diagonal matrix of eigenvalues λ_1_, λ_2_, …, λ*_L_*. Next, define an orthogonal matrix **H = QΛ**^−1/2^**Q***^T^* and **y***_e_* = **H***^T^* **y**. P-values are decorrelated as 1-Φ^−1^(**y***_e_*). Then, the first *k* smallest decorrelated P-values can be used to calculate various combined statistics. The choice of this particular transformation is motivated by its “invariance to order” property. Briefly, in the equicorrelation case, including the special case of *ρ* = 0, i.e., independence, a permutation of **y** should yield the same (possibly permuted) values in the decorrelated vector, **y***_e_*. Extensive evaluation of the decorrelation approach is presented by us elsewhere (Vsevolozhskaya et al., 2019).

## Results

### Simulation Study Results

We used simulation experiments to evaluate the type I error rate and power of the proposed methods relative to the previously studied RTP (defined for a fixed *k*) and to the adaptive RTP [where *k* is varied and the distribution is evaluated by single-layer simulations as in Yu et al. (2009)]. Briefly, a single-layer simulation is a numerical optimization of the naive simulation setup, which is quite slow because each round of data generation has to be followed by a second set of simulations to determine the optimal truncation point. Yu *et al*. proposed a clever shortcut based on a rectangular matrix, in which each *i*-th row is a set of *L* P-values that corresponds to the *i*-th simulation. The shortcut avoids nested and slow simulations but has a downside of keeping quite large matrix in computer memory.

Performance of various methods was evaluated using *k* first-ordered P-values, with *k* = {10,100}, *L* = {100,200,500}, and *B* = 100,000 simulations for evaluation of both, type-I error and power. Details of the simulation design are given in **Supplementary Material S-5**.

When various combined P-value methods are being compared, it is meaningful to gauge their performance against methods designed for multiple testing adjustments. This is especially relevant with methods that employ truncation due to their emphasis on small P-values. Therefore, we included the Simes (1986) method in our power comparisons because it can be viewed as a combined P-value method. The Simes method tests the overall *H*_0_ without a reference to individual P-values: the *H*_0_ is rejected at α level if *P*_(_*_i_*_)_ ≤ *i*α/*L* for at least one *i*. Equivalently, the overall (or the “combined”) Simes P-value can be obtained as min {*kp*_(_*_i_*_)_/*i*}. The Simes test is a useful benchmark, because it is related to the combined P-value methods with truncation, as well as to multiple testing adjustment procedures. At the extreme, the RTP with *k* = 1 becomes equivalent to Šidák (1967) correction. Šidák correction is approximately the Bonferroni (1935) correction for small P-values and large *L*. The Simes P-value is at least as small as Bonferroni-corrected P-value. In addition, there is a connection of the Simes test to the Benjamini and Hochberg (1995) false discovery rate (FDR), i.e., the Simes test is algebraically the same procedure as the Benjamini and Hochberg FDR, although the interpretation is different: FDR method determines the largest *i*, such that *P*_(_*_i_*_)_ ≤ *i*α/*L*, and rejects *H*_0_ for all *P*_(_*_j_*_)_, *j* ≤ *i*, to control the expectation of FDR.

**Tables 1**–**3** present type I error rates for combinations of independent and decorrelated P-values, respectively. In the tables, rows labeled “ART-A” refer to our newly proposed adaptive ART method, while “aRTP” rows label the results of the conventional approach (Yu et al., 2009). For the adaptive methods, the sequence of truncation points varied from 1 to *k* or from 1 to *L*, if *k* = *L*. Both tables confirm that all methods maintain the correct Type I error rate at various α-levels.

**Table 1:** Type I error at α = 0.05, 0.01, and 0.005, assuming that P-values are independent.

	k = 10			k = 100		
	*L* = 100	*L* = 200	*L* = 500	*L* = 100	*L* = 200	*L* = 500
α = 0.05						
RTP	0.0499	0.0502	0.0515	0.0494	0.0511	0.0515
ART	0.0501	0.0504	0.0511	0.0486	0.0509	0.0503
aRTP	0.0499	0.0501	0.0504	0.0510	0.0502	0.0506
ART-A	0.0504	0.0503	0.0507	0.0495	0.0509	0.0489
Simes	0.0499	0.0496	0.0495	0.0507	0.0506	0.0501
α = 0.01						
RTP	0.0106	0.0097	0.0099	0.0100	0.0097	0.0095
ART	0.0102	0.0100	0.0099	0.0103	0.0095	0.0095
aRTP	0.0101	0.0098	0.0101	0.0102	0.0098	0.0095
ART-A	0.0103	0.0103	0.0097	0.0102	0.0105	0.0101
Simes	0.0098	0.0101	0.0099	0.0097	0.0104	0.0091
α = 0.005						
RTP	0.0049	0.0054	0.0046	0.0046	0.0049	0.0044
ART	0.0050	0.0052	0.0050	0.0048	0.0051	0.0046
aRTP	0.0050	0.0049	0.0052	0.0050	0.0049	0.0050
ART-A	0.0049	0.0051	0.0051	0.0050	0.0048	0.0044
Simes	0.0051	0.0050	0.0052	0.0053	0.0048	0.0053

**Table 2:** Type I error at α = 0.05, 0.01, and 0.005 for randomly correlated P-values.

	*L* = *k* = 4	*L* = *k* = 6	*L* = *k* = 10	*L* = 100, *k* = 10	*L* = *k* = 100
Mean | ρ|	0.2924	0.3715	0.4178	0.4634	0.4634
α = 0.05					
RTP	0.0463	0.0495	0.0501	0.0538	0.0541
RTP (decorr)	0.0492	0.0480	0.0515	0.0528	0.0515
ART (decorr)	0.0504	0.0474	0.0501	0.0514	0.0514
aRTP	0.0466	0.0508	0.0489	0.0538	0.0552
ART-A (decorr)	0.0492	0.0500	0.0519	0.0510	0.0543
Simes	0.0516	0.0500	0.0494	0.0510	0.0506
α = 0.01					
RTP	0.0098	0.0099	0.0105	0.0103	0.0096
RTP (decorr)	0.0107	0.0096	0.0095	0.0092	0.0100
ART (decorr)	0.0106	0.0094	0.0101	0.0096	0.0101
aRTP	0.0103	0.0100	0.0097	0.0100	0.0105
ART-A (decorr)	0.0103	0.0100	0.0097	0.0100	0.0105
Simes	0.0101	0.0100	0.0096	0.0099	0.0100
α = 0.005					
RTP	0.0050	0.0048	0.0051	0.0052	0.0052
RTP (decorr)	0.0047	0.0048	0.0056	0.0046	0.0050
ART (decorr)	0.0050	0.0046	0.0056	0.0048	0.0050
aRTP	0.0052	0.0047	0.0050	0.0051	0.0053
ART-A (decorr)	0.0049	0.0048	0.0051	0.0050	0.0049
Simes	0.0051	0.0051	0.0050	0.0048	0.0047

**Table 3:** Additional type I error results at α = 0.05, 0.01, and 0.005 for correlated P-values and different *k* and *L* combinations.

	k = 10			k = 100		
	*L* = 100	*L* = 200	*L* = 500	*L* = 100	*L* = 200	*L* = 500
Mean ρ	0.4634	0.4655	0.4667	0.4655	0.4635	0.4667
α = 0.05						
RTP	0.0538	0.0541	0.0498	0.0509	0.0499	0.0502
RTP (decorr)	0.0528	0.0515	0.0503	0.0497	0.0504	0.0494
ART (decorr)	0.0514	0.0514	0.0501	0.0505	0.0503	0.0498
ART-A	0.0538	0.0552	0.0497	0.0509	0.0497	0.0502
ART-A (decorr)	0.0510	0.0543	0.0498	0.0511	0.0492	0.0505
Simes	0.0510	0.0506	0.0504	0.0514	0.0500	0.0502
α = 0.01						
RTP	0.0103	0.0096	0.0107	0.0096	0.0102	0.0101
RTP (decorr)	0.0092	0.0101	0.0098	0.0100	0.0103	0.0098
mRTP (decorr)	0.0096	0.0100	0.0096	0.0101	0.0101	0.0101
ART	0.0104	0.0098	0.0103	0.0098	0.0094	0.0099
ART-A (decorr)	0.0100	0.0099	0.0098	0.0105	0.0101	0.0103
Simes	0.0099	0.0103	0.0101	0.0100	0.0101	0.0095
α = 0.005						
RTP	0.0052	0.0047	0.0052	0.0052	0.0052	0.0050
RTP (decorr)	0.0046	0.0048	0.0050	0.0050	0.0045	0.0050
mRTP (decorr)	0.0048	0.0050	0.0049	0.0050	0.0047	0.0051
ART	0.0051	0.0045	0.0050	0.0053	0.0050	0.0051
ART-A (decorr)	0.0050	0.0047	0.0046	0.0049	0.0052	0.0054
Simes	0.0048	0.0050	0.0048	0.0047	0.0048	0.0045

**Tables 4**–**6** summarize a set of power simulations for independent P-values. Results presented in **Table 4** were obtained under the assumption that all *L* statistics had the same underlying effect size (*μ* = 0.5). From this table, it is evident that our ART has the highest power, closely followed by RTP. In general, the ART P-values tend to be similar to the P-values obtained by the RTP, and we show their similarity graphically in **Figure 2**. The Simes method has the lowest power, which is expected due to homogeneity in effect sizes across *L* tests and absence of true nulls. For the results in **Table 5**, the effect size was allowed to randomly vary throughout the range from 0.05 to 0.45. In both of these tables, the ART method has the highest power, while the Simes method has the lowest power. The power of both adaptive methods is very similar to one another but lower than that of methods based on a fixed *k* (RTP and ART). Nonetheless, in practice, a good choice for *k* may not be immediately clear, so a small sacrifice in power may be preferable to an arbitrary and possibly poor choice of *k*. However, when *L* is large, it can be impractical or unreasonable to vary candidate truncation points all the way up to *L*. Therefore, the value of *k* in these tables is the upper bound for sets of candidate truncation points, and that explains the difference in powers. For example, for the combination *k* = 10, *L* = 200, the power of ART-A in **Table 5** is 0.26, while for *k* = 100, *L* = 200, the power is 0.37. Adaptive methods are considerably slower than methods where the value of *k* is fixed. A promising solution, which is currently under development, is removal of candidate truncation points for which the corresponding partial products are highly correlated.

**Table 4:** Power under the alternative hypothesis, assuming independence and the same effect size *μ* = 0.5 for all *L* tests.

	k = 10			k = 10		
	*L* = 100	*L* = 200	*L* = 500	*L* = 100	*L* = 200	*L* = 500
RTP	0.35	0.43	0.54	0.49	0.73	0.94
ART	0.38	0.49	0.63	0.50	0.74	0.95
aRTP	0.27	0.33	0.41	0.41	0.61	0.86
ART-A	0.32	0.38	0.46	0.40	0.57	0.72
Simes	0.14	0.16	0.17	0.15	0.16	0.17

**Table 5:** Power under the alternative hypothesis, assuming independence and random effect size (*μ* between 0.05 and 0.45).

	k = 10			k = 10		
	*L* = 100	*L* = 200	*L* = 500	*L* = 100	*L* = 200	*L* = 500
RTP	0.23	0.28	0.37	0.30	0.46	0.72
ART	0.25	0.32	0.43	0.30	0.46	0.75
aRTP	0.18	0.22	0.27	0.25	0.36	0.57
ART-A	0.22	0.26	0.33	0.25	0.37	0.53
Simes	0.12	0.13	0.14	0.12	0.13	0.14

**Table 6:** Power under independence, assuming constant effect size (*μ* = 1.4) for a fraction of *L* = 1,000 hypotheses and *μ* = 0 for the rest of the tests. Proportion of true effects is the proportion of SNPs with *μ*≠0. In other words, it is the proportion of alternative hypotheses among all hypotheses.

	k = 10			k = 50		
Proportion of true effects	2.5%	5%	10%	2.5%	5%	10%
RTP	0.24	0.48	0.83	0.29	0.65	0.97
ART	0.24	0.52	0.89	0.29	0.66	0.98
aRTP	0.20	0.40	0.75	0.25	0.55	0.93
ART-A	0.22	0.45	0.75	0.26	0.56	0.88
Simes	0.14	0.23	0.38	0.14	0.23	0.38

We note that power values in **Tables 4**–**5** include *k* = *L*, but for illustrative purposes, we also give power values for some choices where *k* < *L*. We emphasize that, in our tables, these chosen maximum *k* values do not have any specific meaning. In general, the maximum value of *k* for the adaptive methods can be varied up to *L*, unless *L* is either very large, or if there is *a priori* assumption that the real signals are rare. As discussed in Zaykin et al. (2007a), the optimal value of *k* is usually lower than the actual number of real signals; however, the upper bound for *k* can be set to reflect *a priori* knowledge about potential maximum number of real signals.

Finally, **Table 6** summarizes results for simulations when some of the *L* hypotheses were true nulls (*μ* = 0), while the remaining hypotheses were true signals (*μ* = 0.5). The results follow the same pattern as in the previous tables, with ART having the highest power.

**Table 7** summarizes a set of power simulations for correlated P-values. The effect sizes were randomly varied between −0.45 and 1.3 in each simulation. The correlation matrices were generated as described in **Supplementary Material S-5**. This set of simulations assumes that the P-values were obtained from the same data set as the sample estimate of the correlation matrix. Under heterogeneous effect sizes (**Table 7**), the empirical versions of the tests (“RTP,” “ART-A”) show nearly identical (and low) power for various combinations of *k* and *L* values. However, decorrelation-based methods become quite powerful, and their power is increasing with *k* and *L*. The steady power increase is due to the decorrelation effect on the combined noncentrality that involves the sum $\sum_{i\neq j}^{L}{\left(\mu_{i}-\mu_{j}\right)}^{2}$ , which increases with the increased heterogeneity of *μ*. More details on the performance of the decorrelation approach are given by us elsewhere (Vsevolozhskaya et al., 2019), but here we briefly note that this finding is practically relevant because substantial heterogeneity of associations is expected among genetic variants, leading to a substantial power boost, as we next illustrate *via* re-analysis of published associations of genetic variants within the *μ*-opioid gene with pain sensitivity.

**Table 7:** Power for correlated P-values when the effect size is randomly varied between −0.45 and 1.3.

	*L* = *k* = 4	*L* = *k* = 6	*L* = *k* = 10	*L* = 100, *k* = 10	*L* = *k* = 100
Mean | ρ|	0.39	0.43	0.45	0.47	0.47
RTP	0.13	0.12	0.11	0.17	0.12
RTP (decorr)	0.41	0.47	0.57	0.98	> 0.99
ART (decorr)	0.41	0.47	0.57	0.99	> 0.99
aRTP	0.17	0.16	0.16	0.20	0.18
ART-A (decorr)	0.38	0.44	0.52	0.94	0.98
Simes	0.35	0.38	0.41	0.63	0.64

### Real Data Analysis

In several popular variations of the gene-based approach (Neale and Sham, 2004), future association statistics or P-values are combined across variants within a gene (Yu et al., 2009; Liu et al., 2010; Peng et al., 2010; Li et al., 2011). Gene-based approaches have some advantages over methods based on individual SNPs or haplotypes. In particular, gene-based P-values may facilitate subsequent meta-analysis of separate studies and can be less susceptible to erroneous findings (Neale and Sham, 2004). To obtain a gene-based P-value, one would need to account for LD among variants. The matrix of LD correlation coefficients can be obtained conveniently without access to individual genotypes if frequencies of haplotypes for SNPs within the genetic region of interest are available. The LD for alleles *i* and *j* is defined by the difference between the di-locus haplotype frequency, *P_ij_*, and the product of the frequencies of two alleles, *D_ij_* *_=_* *P_ij_- p_i_p_j_*. The LD correlation for SNPs *i* and *j* is $r_{ij}=\frac{D_{ij}}{p_{i}(1-p_{i})p_{j}(1-p_{j})}$ . Shabalina et al. (2008) and Kuo et al. (2014) reported SNP-based P-values (**Table 8**), as well as results of several haplotype-based tests for genetic association of variants within the μ-opioid receptor (*MOR*) with pain sensitivity. Kuo et al. (2014) also reported estimated frequencies for 11-SNP haplotypes within the *MOR* gene, from which the 11×11 LD correlation matrix can be computed. The *P_ij_* frequencies are given by the sum of frequencies of those 11-SNP haplotypes that contain both of the minor alleles for SNPs *i* and *j*. Similarly, *p_i_* allele frequency is the sum of haplotype frequencies that carry the minor allele of the SNP *i*. The LD correlations within the *MOR* region spanned by the 11 SNPs ranged from −0.82 to 0.99, with the average absolute value ≈0.55 and the median absolute value ≈0.66. Half of pairwise LD correlations were smaller than −0.23 or larger than 0.82. Our analysis (**Table 9**) showcases utility of the proposed methods. The columns show combined P-values, for *k* varying from 2 up to all 11 SNPs (*k* = 1 is equivalent to the Bonferroni correction, i.e., 0.007×11). Similar to what we found *via* simulation experiments, where correlation is controlled by reshuffling the phenotype values while keeping the original LD structure intact, RTP and aRTP (without the decorrelation step) do not benefit from inclusion of additional SNPs. P-values in the ART column are very similar to those in the RTP column, which reassures our theoretical expectations. In contrast to previously proposed methods that control correlation by resampling (i.e., RTP, aRTP, and ART), the results in columns marked by “decorr” are substantially lower. In these columns, we used the proposed transformation to independence, which gives much stronger combined P-values. In all “decorr” columns, *k* = 7 results in the smallest combined P-value, implying that the number of real effects (including proxy associations) is at least seven.

**Table 8:** Individual SNP P-values as originally reported in Shabalina et al. (2008).

SNP	P-value
rs563649	0.0007
rs9322446	0.0941
rs2075572	0.2957
rs533586	0.7037
rs540825	0.8171
rs675026	0.8012
rs660756	0.5745
rs677830	0.9891
rs623956	0.8308
rs609148	0.8208
rs497332	0.3139

**Table 9:** Combined P-values by different methods for *μ*-opioid data.

k	RTP	RTP (decorr)	ART (decorr)	aRTP	ART-A (decorr)
2	0.0187	0.0225	0.0256	0.0519	0.0533
3	0.0411	0.0234	0.0253	0.2963	0.0211
4	0.0566	0.0192	0.0183	0.1845	0.0115
5	0.0886	0.0211	0.0231	0.4702	0.0165
6	0.1172	0.0204	0.0208	0.6543	0.0070
7	0.1486	**0.0177**	**0.0169**	0.7718	**0.0041**
8	0.1726	0.0211	0.0220	0.7189	0.0416
9	0.1810	0.0228	0.0232	0.6766	0.0165
10	0.1867	0.0241	0.0241	0.6423	0.0096
11	0.1938	0.0241	0.0243	0.6140	0.0065

The smallest P-values in decor columns are highlighted in bold. The table reports 11 aRTP values rather than a single optimal one, because one can specify the largest k value, which was varied here from 2 to 11.

## Discussion

Complex diseases are influenced by collective effects of environmental exposures and genetic determinants. There can be numerous weak but biologically meaningful risk factors. The challenge is to distinguish between real and spurious statistical signals in the presence of multiple comparisons and low detection power. When the number of potential real associations is expected to be small, compared to the total number of variants evaluated within a study, it is advantageous to focus on the top-ranking associations. The rank truncated product method (RTP) has been designed with this objective in mind. The RTP and related approaches had been shown to be valuable tools in analysis of genetic associations with disease. In this article, we derive a mathematically simple form of the RTP distribution that leads a to new method, ART and its adaptive version, ART-A, which searches through a number of candidate values of truncation points and finds an optimal number in terms of combined P-value. Two important questions are the meaning of “*k*” and its optimal value. Unfortunately, *k* cannot be interpreted as an estimate of the number of real signals, for the reason that the *k* value that yields the minimum P-value depends on both the numbers of real signals, as well as on their strength of association, both of which are unknown. This issue has been investigated in detail in Zaykin et al. (2007a). The ART is designed with the same objectives in mind as RTP and TPM: to facilitate detection of possibly weak signals among top-ranking predictors that could have been missed, unless combined into a single score. The ART is trivial to implement in terms of standard functions, provided by packages such as R, and its power characteristics are close to RTP or higher in all studied settings. Analytical forms of ART and ART-A are derived under independence. To accommodate LD, we propose a decorrelation step, by transformation of P-values to independence. Our decorrelation by orthogonal transformation approach (DOT) is analogous to the Mahalanobis transformation (Härdle and Simar, 2007). We found DOT to be surprisingly powerful in many settings, compared to the usual method of evaluating the distribution of product of correlated P-values under the null hypothesis. Theoretical properties and extensive numerical evaluation of DOT will be published elsewhere and currently these findings are available as a preprint (Vsevolozhskaya et al., 2019). Further, we illustrate an application of our methods with analyses of variants within the *μ*-opioid gene that have been shown to affect sensitivity to pain. We find strengthened evidence of overall association within the 11-SNP block. In this application, the LD correlation matrix was reconstructed from the haplotype frequencies, which might be slightly different from the correlation of (0,1,2) values between pairs of SNPs (Zaykin, 2004). Further studies are needed to investigate whether approaches such as this, or utilization of reference panel (external) data as a source of LD information, may lead to substantial bias.

## Web Resources

The URL for software referenced in this article is available at: https://github.com/dmitri-zaykin/Total_Decor.

## Data Availability Statement

The datasets generated for this study are available on request to the corresponding author.

## Author Contributions

DZ and OV conceived the study, interpreted the results, and wrote the manuscript. FH performed extensive simulations. All authors read and approved the final manuscript.

## Conflict of Interest

The authors declare that the research was conducted in the absence of any commercial or financial relationships that could be construed as a potential conflict of interest.
