# Supplementary material for: Detecting Weak Signals by Combining Small P-Values in Genetic Association Studies
Source: Front Genet. 2019 Nov 20;10:1051. doi: 10.3389/fgene.2019.01051 (PMC6879667; doi:10.3389/fgene.2019.01051)
Supplement: Supplementary file 1 [file DataSheet_1.pdf]

Supplementary Material

S-1 CORRELATION AND DEPENDENCIES AMONG K SMALLEST P-VALUES

The complexity of analytic forms of the RTP distribution is due to dependency introduced by ordering of P-values. Although order statistics are correlated, products and sums are oblivious to the order of the terms, therefore for the case when $k = L$, the statistic T_k follows the gamma distribution with the shape parameter equal to L, and the unit scale, i.e., $T_L \sim \text{Gamma}(L, 1)$. This is essentially the same as the Fisher combined P-value, where the statistic is $2T_L$, distributed as the chi-square with $2L$ degrees of freedom. However, for $1 \leq k < L$, the k smallest P-values remain dependent even if these k values are not sorted (e.g., randomly shuffled). The dependency is induced through $P_{(k+1)}$ being a random variable: when $P_{(k+1)}$ happens to be relatively small, the k P-values have to squeeze into a relatively small interval from zero to that value. This induces a positive correlation between random sets of k smallest P-values, similar to the clustering effect in the random effects models.

The k smallest unordered P-values are equicorrelated and also have the same marginal distribution, which can be obtained as a permutation distribution of the first k uniform order statistics. Assuming independence of L P-values and their uniform distribution under the null hypothesis, we can derive the correlation between any pair of *unordered* k smallest P-values as $\rho(k, L) = 3(L - k)/(2 + k(L - 2) + 5L)$. As L increases, the correlation approaches the limit that no longer depends on L: $\lim_{L\to\infty} \rho(k, L) = 3/(k + 5)$. The correlation can be substantial for small k and cannot be ignored. There is a very simple transformation that makes a set of k P-values uncorrelated. All that is needed to decorrelate these P-values is to scale the largest of them:

$$
\begin{aligned}
X_1 &= P_{(1)} \\
X_2 &= P_{(2)} \\
&\vdots \\
X_{k-1} &= P_{(k-1)} \\
X_k &= \sigma P_{(k)},
\end{aligned}
$$

where

$$
\sigma = \frac{2L - k + 3 + \sqrt{(k + 1)(L + 1)(L - k + 1)}}{4 + 2L},
$$

and then randomly shuffle the set $X_1, \ldots X_k$. This scale factor σ can be derived by solving the mixture covariance linear equations induced by the permutation distribution of the first k order statistics. The decorrelated values can be further transformed so that each has the uniform (0,1) distribution marginally:

$$
U_j = \frac{1}{k} \sum_{i=1}^{k} \text{Beta}(X_j;\ i, L - i + 1), \quad j = 1, \ldots, k - 1 \tag{S-1}
$$

$$
U_k = \frac{1}{k} \sum_{i=1}^{k} \text{Beta}(X_k/\sigma;\ i, L - i + 1), \tag{S-2}
$$

where Beta$(x;\ a, b)$ is the CDF of a beta(a, b) distribution evaluated at x. Although the scaling and subsequent shuffle removes the correlation, the values remain dependent, as illustrated in Figure 1.

S-2 DERIVATION OF THE RTP DISTRIBUTION

An intuitive way to understand our derivation of the RTP distribution is through references to simulations. The simplest, brute-force algorithm to obtain the RTP combined P-value is by simulating its distribution directly. If w_k is the product of k actual P-values, one can repeatedly (B times) simulate L Uniform(0,1) random variables U_i, sort them, take the product of k smallest values, and compare the resulting product to w_k. As the number of simulations, B, increases, the proportion of times that simulated values will be smaller than w_k converges to the true combined RTP P-value.

There are several ways to optimize the above simulation scenario with respect to computational complexity. For instance, sets of ordered uniform P-values can be simulated directly using well-known results from the theory of order statistics. Despite the fact that the marginal distribution of ith ordered value is Beta$(i, L-i+1)$, to create the necessary dependency between the ordered P-values, sets of k values have to be simulated in a step-wise, conditional fashion. The minimum value, $P_{(1)}$, can be sampled from Beta$(1, L)$ distribution. Alternatively, using the relationship between beta and Uniform(0,1) random variables, it can be sampled as $P_{(1)} = 1 - U_1^{1/L}$. Next, since the value $P_{(2)}$ cannot be smaller than $P_{(1)} = p_{(1)}$, conditionally on the obtained value, it has to be generated from a truncated beta distribution. The third smallest value should be sampled conditionally on the second one, and so on (Balakrishnan and Rao (1998)). Therefore, the sequence and the product w_k can be obtained by simulating k ordered P-values, rather then all L unsorted values.

$$\begin{aligned} P_{(1)} &= 1 - U_1^{\frac{1}{L}} \\ P_{(2)} &= 1 - u_1^{\frac{1}{L}} U_2^{\frac{1}{L-1}} \\ P_{(3)} &= 1 - u_1^{\frac{1}{L}} u_2^{\frac{1}{L-1}} U_3^{\frac{1}{L-2}} \\ &\vdots \\ P_{(k)} &= 1 - u_1^{\frac{1}{L}} u_2^{\frac{1}{L-1}} \dots U_k^{\frac{1}{L-k+1}}. \end{aligned} \tag{S-3}$$

Further optimization of the simulation algorithm is illustrative because it provides intuition for theoretical derivation of the RTP distribution. This optimization is achieved by using the Markov property of order statistics. Specifically, the unordered set $\{P_1, P_2, \dots, P_k \mid P_{(k+1)} = p_{(k+1)}\}$ behaves as a sample of k independent variables, identically distributed as Uniform $\big(0, p_{(k+1)}\big)$. This is a usual step in analytic derivations of product truncated distributions, and it follows by averaging over the density of $P_{(k+1)}$. This approach was employed earlier by Dudbridge and Koeleman (2003) and our derivation of an alternative form of the RTP distributions follows this conditioning idea. After re-scaling,

$$\left\{ \frac{P_1}{p_{(k+1)}}, \frac{P_2}{p_{(k+1)}}, \dots, \frac{P_k}{p_{(k+1)}} \right\} \sim \text{Unif}(0, 1). \tag{S-4}$$

The capital P_i notation is used here to emphasize the fact that the variable is random, while the lowercase $p_{(k+1)}$ refers to a realized value of a random variable, $P_{(k+1)} = p_{(k+1)}$. Next, given that $P_{(k+1)} \sim$

$\text{Beta}(k+1, L-k)$, minus log of the product of independent conditional uniform random variables will follow a gamma distribution. Specifically,

$$-\ln \prod_{i=1}^{k} \frac{P_i}{p_{(k+1)}} = k \ln p_{(k+1)} - \sum_{i=1}^{k} \ln P_i,$$

and treating $p_{(k+1)}$ as a constant,

$$-\ln \prod_{i=1}^{k} \frac{P_i}{p_{(k+1)}} \sim \frac{1}{2}\chi^2_{2k} = \text{Gamma}(k, 1).$$

The above manipulations reduce the set of k random variables to a set of just two variables: a gamma and a beta. Therefore, the combined RTP P-value can be evaluated numerically by simulating only pairs of beta- and gamma-distributed random variables as follows. We note that

$$-\ln \left(\prod_{i=1}^{k} P_{(i)} \right) = -\ln \left(\prod_{i=1}^{k} \frac{P_i}{p_{(k+1)}} \right) - k \ln p_{(k+1)}, \tag{S-5}$$

and define

$$X = P_{(k+1)} \sim \text{Beta}(k+1, L-k) \tag{S-6}$$

$$Y \mid X = -\ln \left(\prod_{i=1}^{k} \frac{P_i}{p_{(k+1)}} \right) \sim \text{Gamma}(k, 1). \tag{S-7}$$

The empirical distribution of the product of k values under H_0 can then be obtained by repeatedly simulating X and Y, and comparing the observed value of $-\ln(w_k)$ to $Z = Y - k\ln(X)$ in every simulation. P_{RTP} would then be defined as the proportion of times simulated values of Z were larger than $-\ln(w_k)$. Surprisingly, one can simultaneously evaluate probabilities for two consequtive partial products,

$$\Pr(W_k \leq w), \quad \text{and}$$
$$\Pr(W_{k+1} \leq w),$$

by reusing the same pair of random numbers, which follows from the fact that

$$-\ln \left(\prod_{i=1}^{k+1} P_{(i)} \right) = -\ln \left(\prod_{i=1}^{k} \frac{P_i}{p_{(k+1)}} \right) - (k+1) \ln p_{(k+1)}. \tag{S-8}$$

In the latter case, $-\ln(w)$ is compared to $Z = Y - (k+1)\ln(X)$. This simulation method is very fast and approaches the exact solution as the number of simulated pairs increases. Moreover, through these simulation experiments it becomes clear that once one conditions on the observed value of $p_{(k+1)}$, the test statistic is formed as a product/sum of independent random variables. Specifically, Gamma distribution for the Y variable in Eq. (S-7) appears to be conditional on the observed $X = p_{(k+1)}$ when the pairs (X, Y) are simulated. Alternatively, one can first simulate $X = p_{(k+1)}$ and then generate a test statistic using k uniform random variables, $U_1, U_2, \ldots, U_k$, on (0, $p_{(k+1)}$) interval.

We just described a way to evaluate the RTP distribution by repeated sampling of two random variables to elucidate the idea that the combined RTP P-value can be obtained by integrating out the random upper bound $P_{(k+1)}$ over its probability density function. Random $P_{(k+1)}$ has to be at least as large as $p_{(k)}$ but smaller than one, $p_{(k)} \leq P_{(k+1)} \leq 1$. After re-expressing $p_{(k)}$ in terms of the observed product $w = \prod_{i=1}^{k} p_{(i)}$, it becomes evident that $w^{1/k} \leq P_{(k+1)} \leq 1$ because the product is maximized if $p_{(i)} = p_{(k)}$ for all $i = 1, \ldots, k$, so the observed $p_{(k)}$ can be at most $w^{1/k}$. Now, integrating over the Beta density, $f(\cdot)$ with parameters $k+1, L-k$, of a single variable $P_{(k+1)}$, we will treat w as a constant:

$$\Pr(W_k \leq w) \quad = \quad 1 - \int_{w^{1/k}}^{1} G_k \left\{ \ln \left(\frac{t^k}{w} \right) \right\} f(t) dt. \tag{S-9}$$

Next, following a transformation, we can express the integral as an expectation and make the integration limits to be 0 to 1, and thus, independent of k:

$$P_{\text{RTP}}(k) = \Pr(W_k \leq w) = 1 - \int_{0}^{1} G_k \left\{ \ln \left(\frac{\left[B_{k+1}^{-1}(u)\right]^k}{w} \right) \right\} du, \tag{S-10}$$

where $B_{k+1}^{-1}(\cdot)$ is inverse CDF of Beta$(k+1, L-k)$ distribution, and $G_k(\cdot)$ is CDF of Gamma$(k, 1)$. $P_{\text{RTP}}(k)$ is the combined RTP P-value. Also note that two partial products can be evaluated at the same time,

$$\Pr(W_{k+1} \leq w) \quad = \quad 1 - \int_{0}^{1} G_k \left\{ \ln \left(\frac{\left[B_{k+1}^{-1}(u)\right]^{k+1}}{w} \right) \right\} du. \tag{S-11}$$

We have now derived simple expressions that involve only a single integral where the integration limits (Eq. (S-10)) no longer involve a product value w and are conveniently bounded within zero to one interval. Eq. (S-10) illustrates that the RTP distribution can be viewed as the expectation of a function of a uniform random variable, U ~Uniform(0,1). If we let $H(u \mid k, w) = G_k \left(\ln \left(\frac{\left[B_{k+1}^{-1}(u)\right]^k}{w} \right) \right)$, the unconditional distribution of W_k is

$$\Pr(W_k \leq w) = 1 - \int_{0}^{1} H(u \mid k, w) du = 1 - E\left\{H(U \mid k, w)\right\}.$$

Therefore, to evaluate $P_{\text{RTP}}(k)$ numerically, one can simply sample a large number of uniform random numbers, U, apply the function $1 - H(U)$ and then take the mean. The corresponding R code using one million random numbers is:

```
mean(1-pgamma(log(qbeta(runif(1e6),k+1,L-k))*k+z,k))
```

where z $= -\ln(w)$. Using the integration explicitly, the R code is:

```
integrate(function(x,w,k,L) 1-pgamma(log(qbeta(x,k+1,L-k))*k+w,k),0,1,z,k,L)$va.
```

S-3 R CODE EXAMPLE FOR COMPUTATION OF ART AND P_{RTP}

In the code below, ART and P_{RTP} are computed for a vector of six P-values with $k = 4$, `lW`$= \sum_{i=1}^{k-1} \ln(P_{(i)})$ and `Pk`$= P_{(k)}$:

```
Art <- function(lW, Pk, k, L) {
   d = (k-1)*(digamma(L+1) - digamma(k))
   ak = (k-1)*log(Pk) - lW + qgamma(1-pbeta(Pk, k, L-k+1), shape=d)
   1 - pgamma(ak, shape=k+d-1)
}
P = sort(c(0.7, 0.07, 0.15, 0.12, 0.08, 0.09))
L = length(P)
k = 4
Z = sum(-log(P[1:k]))
lW = sum(log(P[1:(k-1)]))
P.rtp = integrate(function(x,y,m,n) 1-pgamma(log(qbeta(x,m+1,n-m))*m+y,m),0,1,Z,k,L)
P.ak = Art(lW, P[k], k, L)
```

The resulting combined P-values are ART=0.045 and P_{RTP}=0.047. Note that all six original P-values are larger than the combined ART and RTP. This example demonstrates that weak signals can form a much stronger one after they are combined.

S-4 DERIVATION OF THE ART-A DISTRIBUTION

As we discussed, ordered P-values can be represented as functions of the same number of independent uniform random variables (Eq. S-3). This reveals that the jth value, $p_{(j)}$, is a function of all $p_{(i<j)}$ and that in a given set of k variables (i.e., conditionally) all information is contained in k independent random variables, $U_1, U_2, \ldots, U_k$. These independent components can be extracted and utilized. Specifically, by using the conditional distribution of W_i, which only depends on the two preceding partial products, W_{i-1} and W_{i-2}, we define independent variables Z_i's as $Z_i = \Pr(W_i > w_i \mid W_{i-1}, W_{i-2})$. Successive partial products relate to one another as:

$$W_k = W_{k-1} - W_{k-1}\left(1 - \frac{W_{k-1}}{W_{k-2}}\right) U_k^{\frac{1}{L-k+1}}.$$

Since $U^{\frac{1}{L-k+1}} \sim \text{Beta}(L-k+1, 1)$, the conditional density and the CDF for the product are found as follows:

$$f(W_k = x \mid W_{k-1} = t_{k-1}, W_{k-2} = t_{k-2}) = \frac{(t_{k-1} - x)^{L-k}}{B(L-k+1,1)\left(t_{k-1}(1 - \frac{t_{k-1}}{t_{k-2}})\right)^{L-k+1}}.$$

Let

$$\begin{aligned}
1 - Z_i &= \Pr\left(W_i < w_i \mid W_{k-1} = t_{k-1}, W_{k-2} = t_{k-2}\right) \\
&= \Pr\left(t_{i-1} - t_{i-1}\left(1 - \frac{t_{i-1}}{t_{i-2}}\right) U_i^{\frac{1}{L-i+1}} < w_i \mid W_{i-1} = t_{i-1}, W_{i-2} = t_{i-2}\right) \\
&= \Pr\left(-\ln U_i < -(L-i+1)\ln\left(\frac{1-p_{(i)}}{1-p_{(i-1)}}\right)\right).
\end{aligned}$$

Then,

$$\begin{aligned}
\Pr(W_k \le x \mid W_{k-1} = t_{k-1}, W_{k-2} = t_{k-2}) &= \int_{t_{k-1}^2/t_{k-2}}^{x} f(W_k = x \mid W_{k-1} = t_{k-1}, W_{k-2}) dx \\
&= \frac{1}{B(L-k+1,1)\left(t_{k-1}\left(1-\frac{t_{k-1}}{t_{k-2}}\right)\right)} \\
&\times \int_{t_{k-1}^2/t_{k-2}}^{x} (t_{k-1}-x)^{L-k} dx \\
&= 1 - \left(\frac{t_{k-1}-x}{t_{k-1}\left(1-\frac{t_{k-1}}{t_{k-2}}\right)}\right)^{L-k+1} \\
&= 1 - \left(\frac{1-p_{(k)}}{1-p_{(k-1)}}\right)^{L-k+1} .
\end{aligned}$$

We now obtained a transformation to a new set of independent uniform $(0-1)$ random variables.

$$Z_i = \left(\frac{1-p_{(i)}}{1-p_{(i-1)}}\right)^{L-i+1},$$

with

$$Z_1 = \left(1-p_{(1)}\right)^L .$$

Next, define $Y = \sum_{i=1}^{k} G_{\lambda_i}^{-1}(1-Z_i)$, where $G_{\lambda_i}^{-1}$ is the inverse gamma CDF with the shape λ_i and the scale 1. Under H_0, Y has a gamma distribution with the shape equal to the sum: $\sum_{i=1}^{k} \lambda_i$. The combined P-value is now obtained as:

$$1 - G_{\sum_{i=1}^{k}\lambda_i}\left(\sum_{i=1}^{k} G_{\lambda_i}^{-1}(1-Z_i)\right). \tag{S-12}$$

When λ_i is large, the gamma CDF approaches the standard normal CDF, which motivates the inverse normal transformation. The quantiles will be calculated by using $\lambda_i \Phi^{-1}(1-Z_i)$, as an approximation to $G_{\lambda_i}^{-1}(1-Z_i)$ for large k. The inverse normal method is useful for the reason that the joint distribution of the partial sums can be derived in a standard way to evaluate the adaptive ART (ART-A) P-value. For the ART-A, we define partial sums as:

$$S_k = \sum_{i=1}^{k} \lambda_i \Phi^{-1}(1-Z_i),$$

where $\Phi^{-1}(\cdot)$ is inverse CDF of the standard normal distribution. Then, under the null hypothesis, $\mathbf{S} = (S_1, S_2, ..., S_k)^T$ follows a multiviate normal distribution, MVN$(\mathbf{0}, \mathbf{\Sigma})$, with $\mathbf{\Sigma} = \mathbf{FWF}^T$ and

$$\mathbf{F} = \begin{bmatrix} 1 & 0 & \cdots & 0 & 0 \\ 1 & 1 & \cdots & 0 & 0 \\ \vdots & \vdots & \ddots & \vdots & \vdots \\ 1 & 1 & \cdots & 1 & 0 \\ 1 & 1 & \cdots & 1 & 1 \end{bmatrix}, \ \mathrm{diag}(\mathbf{W}) = \begin{bmatrix} \lambda_1^2 & & & \\ & \lambda_2^2 & & \\ & & \ddots & \\ & & & \lambda_k^2 \end{bmatrix},$$

where λ are weights. In our simulation experiments, we set all $\lambda_i = 1$, however one may take advantage of some information about the effect size distribution, if that is available. If power is high, but the signal is sparse, it would be expected that true signals may tend to rank among the smallest P-values. In this case, one possible sequence of weights is $\lambda_{k-i+1}^2 = \frac{k}{k-i+1}$. Such weights that emphasize partial sums with few terms can also be used in certain situations where P-value distribution is expected to be skewed from the uniform (e.g., due to discreteness of a test statistic), with many P-values being close to one. Finally, the vector $\mathbf{S}$ can be standardized as $T_i = S_i/\sigma_i$, where σ_i are the diagonal elements of $\mathbf{\Sigma}$, then $\mathbf{T} \sim \mathrm{MVN}(\mathbf{0}, \mathbf{R})$, $R_{ij} = \frac{\Sigma_{ij}}{\sqrt{\Sigma_{ii}\Sigma_{jj}}}$. The null distribution of $\mathbf{T}$ is used to evaluate ART-A by using $\Pr(S_i/\sigma_i > s_i)$ probabilities and to obtain quantiles (significance thresholds) using commonly available MVN distribution functions (e.g., `mvtnorm` R package) (Mi et al. (2009)).

S-5 SIMULATION SETUP

We performed B=100,000 simulations to evaluate the Type I error rate and power of the proposed methods. To study performance of combination methods for independent P-values, in each simulation, we generated L normally distributed statistics, $X \sim N(\mu, 1)$. The squared values of X follow the chi-square distribution with one degree of freedom and noncentrality parameter μ^2, $X^2 \sim \chi^2_{(1,\mu^2)}$. P-values were obtained as one minus the CDF of the noncentral chi-square evaluated at X^2, or as $P = 2 - \Phi\left(|X| + |\mu|\right) - \Phi\left(|X| - |\mu|\right)$ in terms of the normal CDF. P-values generated from normal statistics (without squaring them) were also considered, but these results are omitted for brevity, because the resulting ranking of the methods by power was found to be similar. Under H_0, L P-values were sampled from the uniform (0, 1) distribution, which is equivalent to setting μ to zero.

To study non-independent P-values, we simulated L statistics from a mutivariate normal distribution MVN $(\boldsymbol{\mu}, \mathbf{\Sigma})$ and decorrelated them by eigendecomposition as described in "Methods" section. In each simulation, a correlation matrix $\mathbf{\Sigma}$ was generated randomly by perturbing an equicorrelated matrix $\mathbf{D}$. Specifically, we added perturbation to equicorrelated matrix $\mathbf{D}$ with off-diagonal elements $\rho = 0.5$ as:

$$\mathbf{R} = \mathbf{D} + \mathbf{u}\mathbf{u}^T, \qquad \text{(S-13)}$$

where $\mathbf{u}$ is random vector (Bartlett (1951)). Then, $\mathbf{R}$ was converted to a correlation matrix $\mathbf{\Sigma}$ with off-diagonal elements $\rho_{ij} = \frac{R_{ij}}{\sqrt{R_{ii}R_{jj}}} = \frac{\rho + u_i u_j}{\sqrt{1+u_i^2}\sqrt{1+u_j^2}}$. The amount of "jiggle" in $\mathbf{R}$ depends on the variability of elements in $\mathbf{u}$. If elements of $\mathbf{u}$ are generated in the range between $-\delta$ and δ, the value of δ would represent the upper bound for the amount of jiggle allowed between pairwise correlations in $\mathbf{\Sigma}$. In our simulations, we set $\delta = 1$, allowing for a mix of positive and negative values of ρ_{ij} in $\mathbf{\Sigma}$.

In addition, we evaluated power of the methods by using correlation due to linkage disequilibrium (LD) in real data (Table (S1). The 11 $\times$11 correlation matrix was estimated from previously reported haplotype frequencies of eleven SNPs in the μ-opioid receptor (*MOR*) gene (Shabalina et al. (2008); Kuo et al. (2014)).

The pairwise LD correlations within *MOR* were generally high and ranged from -0.82 to 0.99. In this set of simulations, we used effect sizes sampled uniformly in the interval from -0.5 to 0.2.

The Type I error rate and power performance were computed based on two $B \times k$ matrices of P-values, $\mathbf{P}_0$ and $\mathbf{P}_A$, every row of which contained k smallest sorted P-values out of L tests across B simulations ($L - k$ P-values were discarded). $\mathbf{P}_0$ stored simulated P-values under H_0 and $\mathbf{P}_A$ under the alternative hypothesis, H_A. Taking the product of P-values in each row, we obtain two $B \times 1$ vectors, $\mathbf{w}_0$, $\mathbf{w}_A$. RTP P-values were computed based on the empirical CDF (eCDF) of $\mathbf{w}_0$ evaluated at B values of $\mathbf{w}_A$. Power was calculated as the proportion of P-values that were smaller than the significance threshold, α.

$L = 11$, mean $\lvert\rho\rvert = 0.55$					
k:	5	6	7	9	11
RTP	0.07	0.06	0.06	0.05	0.05
RTP(decorr)	0.85	0.87	0.87	0.87	0.87
ART(decorr)	0.85	0.87	0.87	0.87	0.87
aRTP	0.07	0.07	0.06	0.06	0.06
ART-A(decorr)	0.85	0.85	0.85	0.84	0.83
Simes	0.78	0.78	0.78	0.78	0.79

Table S1. Power at $\alpha = 0.05$ for P-values correlated according to the LD structure in the μ-opioid gene, with effect sizes randomly sampled in the interval from -0.5 to 0.2.

REFERENCES

Balakrishnan, N. and Rao, C. R. (1998). *Order statistics: theory & methods* (Elsevier Amsterdam)

Bartlett, M. S. (1951). An inverse matrix adjustment arising in discriminant analysis. *The Annals of Mathematical Statistics* 22, 107–111

Dudbridge, F. and Koeleman, B. P. (2003). Rank truncated product of p-values, with application to genomewide association scans. *Genet Epidemiol* 25, 360–366

Kuo, C.-L., Diatchenko, L., and Zaykin, D. (2014). Discovering multilocus associations with complex pain phenotypes. *Pain Genetics: Basic to Translational Science* , 99–114

Mi, X., Miwa, T., and Hothorn, T. (2009). mvtnorm: New numerical algorithm for multivariate normal probabilities. *The R Journal* 1, 37–39

Shabalina, S. A., Zaykin, D. V., Gris, P., Ogurtsov, A. Y., Gauthier, J., Shibata, K., et al. (2008). Expansion of the human μ-opioid receptor gene architecture: novel functional variants. *Human Molecular Genetics* 18, 1037–1051
